# Analyses of putative anti-cancer potential of three STAT3 signaling inhibitory compounds derived from *Salvia officinalis*

**DOI:** 10.1016/j.bbrep.2020.100882

**Published:** 2020-12-24

**Authors:** Maho Yanagimichi, Katsutoshi Nishino, Akiho Sakamoto, Ryusei Kurodai, Kenji Kojima, Nozomu Eto, Hiroko Isoda, Riadh Ksouri, Kazuhiro Irie, Taiho Kambe, Seiji Masuda, Toru Akita, Kazuhiro Maejima, Masaya Nagao

**Affiliations:** a Graduate School of Biostudies, Kyoto University, Kyoto, 606-8502, Japan; b Graduate School of Agriculture, Kyoto University, Kyoto, 606-8502, Japan; c Interdisciplinary Graduate School of Agriculture and Engineering, University of Miyazaki, Miyazaki, 889-2192, Japan; d Faculty of Life and Environmental Sciences, University of Tsukuba, Ibaraki, 305-8572, Japan; e Alliance for Research on the Mediterranean and North Africa (ARENA), University of Tsukuba, Ibaraki, 305-8572, Japan; f Centre de Biotechnologie à la Technopole de Borj Cédria (CBBC), BP 901, 2050, Hammam-lif, Tunisia; g Nippon Shinyaku CO., LTD., Kyoto, 601-8550, Japan

**Keywords:** *Salvia officinalis* (Common sage), Cirsiliol, STAT3, NK cells, Anti-cancer, SDS, Sodium dodecyl sulphate, PAGE, Polyacrylamide gel electrophoresis, HPLC, High performance liquid chromatography, STAT3, signal transducer and activation of transcription 3, MTT, 3-(4,5-dimethylthiazol-2-yl)-2,5-diphenyltetrazolium bromide, PBS, phosphate buffered saline

## Abstract

The extract of *Salvia officinalis* (Common Sage) exhibited inhibitory activity of STAT3 signal after screening of several plants extracts using the STAT3-responsive reporter system. Cirsiliol, luteolin, and carnosol were identified from the methanol extract of *Silvia officinalis* as inhibitors of STAT3 signaling and the effects of these three compounds on STAT3 protein or growth inhibition on cancer cells was compared. Luteolin at the dose of 90 μM clearly suppressed the phosphorylation of STAT3 induced by IL-6, while carnosol was prone to decrease total STAT3 proteins at high doses (>90 μM). Cirsiliol had almost no effect. Since the three compounds exhibited similar concentration-dependent suppression patterns in the reporter assay except for cirsiliol became plateau beyond 30 μM, these compounds appeared to function as STAT3 inhibitory factors in different ways. The direct anti-proliferative activity of three compounds was examined with or without the anti-cancer drug gefitinib using HepG2 and A549 cells. The anti-proliferative effect of the three compounds was additively enhanced by gefitinib. At the doses of 3.6 μM, statistically significant suppression of proliferation was observed in HepG2 cells only by cirsiliol among the three compounds in the absence of gefitinib but all three compounds were prone to suppress the proliferation of HepG2 cells and A549 cells dose-dependently although cirsiliol showed a modest dose-dependency and this suppression of proliferation was enhanced by the addition of gefitinib. Cirsiliol, a dimethyoxylated flavone, activated the natural killer activity of KHYG-1 cells against erythroleukemia K562 cells like a hexamethoxylated flavone, nobiletin, suggesting that it may also have an indirect anti-cancer potential through activation of NK cells. These results shed light on the putative anti-cancer potential of *Salvia officinalis*.

## Highlights

• Carnosol, luteolin and cirsiliol were identified as STAT3 signal inhibitors in *S. officinalis.*
• Cirsiliol inhibited the STAT3-responsive reporter expression at 7.5 μM but showed low dose-dependency at higher doses.
• Cirsiliol at 90 μM showed almost no effect on phosphorylation of STAT3 and weakly suppressed total STAT3.
• Cirsiliol exhibited anti-proliferative activity at 3.6 μM against HepG2 cells and A549 cells but showed low dose-dependency.
• Cirsiliol activated NK cells by stimulating exocytosis of granules for cytolysis.

## Graphical abstract

**Figure:**
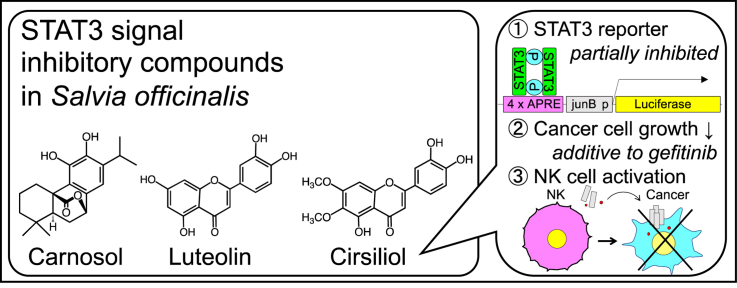

## 1 Introduction

*Salvia officinalis* (Common Sage) is a medicinal or culinary herb [1] originally from the Mediterranean area including north Africa, Spain, France, Italy and the Balkans. The extract of common sage has anti-diabetic, anti-septic, and anti-inflammatory properties [2]. In this study, cirsiliol, luteolin and carnosol were identified as inhibitors of the signal transducer and activator of transcription 3 (STAT3) signaling in the extract of common sage by the use of the STAT3-responsive reporter system. Although suppression of IL-6 dependent- or independent expression of other STAT3-responsive reporter expression by cirsiliol in Hep3B cells or by carnosol in HCT116 cells, respectively [3,4], and luteolin-dependent inhibition of STAT3 activation through disruption of binding of HSP90 to STAT3 in gastric cancer cells have been reported [5], suppression of IL-6-dependent expression of the IL-6 responsive reporter expression and change in the quantity of phosphorylated or total STAT3 proteins by these three compounds simultaneously have not been compared in HepG2 cells. STAT3 is a transcription factor that is activated through cell surface receptors such as the epidermal growth factor receptor and cytokine receptors including an IL-6 receptor complex or a non-receptor tyrosine kinase, such as Src by phosphorylation at a tyrosine residue (Tyr705) and a dimer of phosphorylated STAT3 translocated to the nucleus and functions as a transcription factor to regulate the expression of genes that mediate proliferation, survival, and angiogenesis [6]. In tumors, aberrant STAT3 activation can occur by canonical signaling where STAT3 monomers are phosphorylated at Tyr705 or by a noncanonical pathway or noncanonical pathway of STAT3 signaling where STAT3 is phosphorylated at serine 727 of STAT3 by mitogen-activated protein kinase, c-Jun N-terminal kinase or protein kinase C [7], which suggests that STAT3 is a promising target for cancer therapy [8]. STAT3 inhibitors from natural sources regulate STAT3 signaling by various mechanisms, for example, inhibition of phosphorylation through various signals, dimerization, DNA binding, and degradation of STAT3. Furthermore, STAT3 signaling inhibitors from natural sources may have multiple functions besides inhibition of STAT3 signaling [9]. Gefitinib (Iressa) is a tyrosine kinase inhibitor of epidermal growth factor receptor (EGFR) kinase, but gefitinib resistance resulting from activation of STAT3 signaling has been reported to occur in cancer patients [10,11]. In this study, we examined the additive anti-proliferative effect of gefitinib on cancer cells treated by these three compounds and found that cirsiliol had unique characters in STAT3-responding signals among the three compounds. Further we examined the activation of natural killer cells by cirsiliol, a dimethoxyflavone, since nobiletin, a hexamethoxyflavone was reported to activate cytolytic activity of NK cells [12].

## 2 Materials and methods

### 2.1 Chemicals

Carnosol (Wako), luteolin (Santa Cruz), cirsiliol (Sigma-Aldrich), AG490 (Calbiochem), gefitinib (Cayman), nobiletin (Wako), and apigenin (Wako) were dissolved in DMSO (Nacalai).

### 2.2 Cell culture

HepG2 cells (RIKEN RBC) and A549 cells (JCRB) were cultured in Dulbecco's modified Eagle medium (WAKO). K562 cells (JCRB) and KHYG-1 cells (JCRB) were cultured in RPMI1640 medium (Wako). Both media contained 10% heat-inactivated fetal bovine serum (Biosera), 100 U/ml penicillin and 100 μg/ml streptomycin (Nacalai). KHYG-1 cells were maintained in the presence of 100 IU/mL human recombinant IL-2 (Wako).

### 2.3 Purification of STAT3 signal inhibitors from the extract of *Salvia officinalis*

**Fig. 1:**
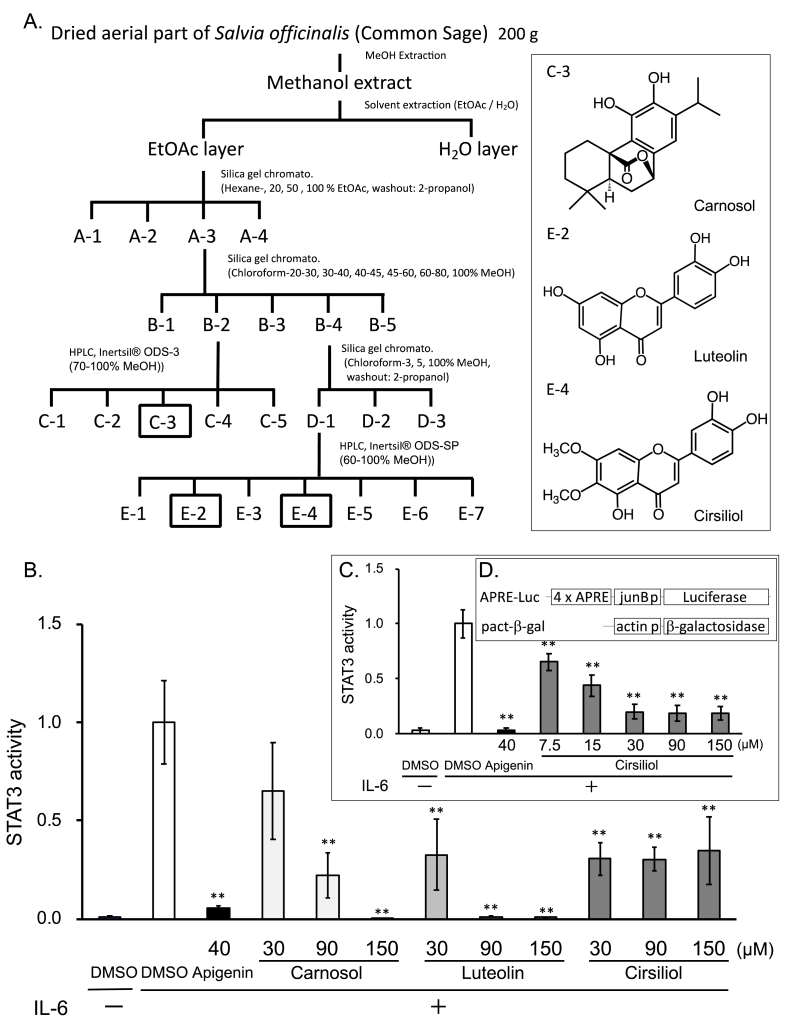
Identification of three compounds as STAT3 signal inhibitors from the methanol extract of *Salvia officinalis*. (A) Purification scheme and identified compounds. (B, C) Estimation of dose-dependent STAT3 signal inhibitory activity by three compounds (B) or cirsiliol (C) using STAT3 reporter assay. Apigenin (40 μM) was employed as positive control. Results are presented as means ± SD (n = 3). STAT3 activity indicated luciferase activity normalized by β-galactosidase activity. *p < 0.05 and **p < 0.01 when compared with IL-6-treated group by Student's *t*-test. (D) Schemes of reporter constructs.

The dried aerial part of *Salvia officinalis* (200 g, K. Kobayashi & Co., Ltd.) was extracted in 1.8 L methanol. Precise purification steps are described in Fig. 1A.

### 2.4 NMR and LC-MS analysis

^1^H NMR spectra were measured and recorded on an Avance III 400 (Bruker, Germany). HR-ESI-MS data were obtained on a Waters ACQUITY™ UPLC with Xevo G2 QTof mass spectrometer (Nihon Waters, Japan). NMR spectra of each compound identified in the extract were confirmed using commercially available compounds and NMR spectra reported on each compound [[13], [14], [15]].

### 2.5 STAT3 reporter assay

The reporter plasmid that contains four copies of acute-phase response elements in front of the minimal junB promoter linked to the firefly luciferase gene (APRE-Luc, Fig. 1D) [16] and pact-βgal reporter carrying the β-galactosidase gene under control of the chicken β-actin promoter (pact-βgal, Fig. 1D) [17] were co-transfected to HepG2 cells by Hily max (Dojindo). Transfected cells (7.0 × 10^3^ cells) seeded on a 96-well plate were cultured for 36 h, and medium was replaced by medium containing IL-6 (final 30 ng/ml, Peprotech) and each test sample. After incubation for 4 h, cells were lysed by 1x Passive lysis buffer (Promega) and luciferase and β-galactosidase activity of the lysate was measured as described previously [18].

### 2.6 MTT assay

A549 and HepG2 cells seeded in 96-well plates (3.0 × 10^3^ cells in 100 μL) were cultured for 24 h and incubated for another 72 h in the presence of test samples. Then 10 μl of 5 mg/ml MTT in PBS was added. After incubation for 4 h, cells were extracted with DMSO and measured for absorbance at 535 nm.

### 2.7 NK assay

NK assay was performed as described previously [19]. Briefly, K562 cells (1 × 10^4^ cells) pre-incubated in assay medium (RPMI1640 without phenol red, 1% BSA) containing 10 μg/ml calcein-AM for 30 min were washed in RPMI1640 and then mixed with KHYG-1 cells (1 × 10^4^ cells) in a 96-well plate with 200 μL of assay medium. After culture for 4 h at 37 °C in 5% CO_2_, fluorescence of calcein in the culture supernatant was measured with Powerscan 4 (Excitation 485 nm/Emission 538 nm, DS pharma). Cytotoxicity was measured; where, Cytotoxicity (%) = [(experimental release - spontaneous release)/(maximum release – spontaneous release)] x 100. Maximal or spontaneous release was determined by solubilizing K562 cells that were treated with calsein-AM in lysis buffer containing 2% Triton X-100 or by estimating calcein in the culture supernatant of K562 cells.

### 2.8 Western blot of STAT3 or perforin and granzyme B

In Western blot analysis of STAT3, HepG2 cells (1.4 × 10^5^ cells/12-well plate) cultured overnight in growth medium were washed with PBS and cultured in DMEM without FBS for 24 h. Then they were cultured in the presence of test samples for 4 h. IL-6 was added (final 60 nM) and after 15 min, cells were lysed in cell lysis buffer (50 mM Tris-HCl(pH7.4), 150 mM NaCl, 1 mM EDTA, 1% Triton-X100, 0.1% SDS, 10 mM NaF, 1 mM NaVO_3_). Each lysate containing 10 μg of proteins after heat treatment at 70 °C for 10 min in 1 x SDS sample buffer (Nacalai) was separated by SDS-PAGE and transferred to Immobilon® -P Transfer Membrane PVDF 0.45 μm (Millipore).

In Western blot analysis of perforin and granzyme B, KHYG-1 cells (1 × 10^5^ cells/12 well plate) were cultured with each test sample for 24 h and cells were lysed in cell lysis buffer (50 mM Tris-HCl(pH7.4), 150 mM NaCl, 1 mM EDTA, 1% Triton-X100, 0.1% SDS, 1% protease Inhibitor Cocktail (Sigma)). Each lysate containing 5 μg of proteins was separated by SDS-PAGE.

Phospho-Stat3 Y705 D3A7 XP® Rabbit mAb (Cell signaling Technology, # 9145, 1:6000), Stat3 124H6 Mouse mAb (Cell signaling Technology, #9139, 1:2000), GAPDH FL-335 rabbit polyclonal IgG (Santa Cruz, sc-25778, 1:6000), Perforin 1 F-1 mouse monoclonal IgG2b (Santa Cruz, sc-136994, 1:1000) and Granzyme B Rabbit Ab (Cell signaling Technology, #4275, 1:2000) were used as 1st antibodies. Horseradish peroxidase-linked anti-rabbit IgG from donkeys or anti-mouse IgG from sheep (GE Healthcare, NA934, 1: 3000, or NA931, 1:3000, respectively) were used as 2nd antibodies. ECL™ Western Blotting Detection System (GE Healthcare) and ImageQuant LAS 500 (GE Healthcare) were used for detection. The intensity of each band was calculated by ImageJ (NIH).

### 2.9 Statistical analysis

All experiments were repeated at least twice. Most data are presented as triplicate and reported as means ± standard deviations unless otherwise stated. Statistical significance was evaluated by either the Student's *t*-test, or Dunnett's test. A value < 0.05 was considered statistically significant.

## 3 Results

We screened the STAT3 signal inhibitors in various plant extracts using the APRE-luc reporter system that responds to IL-6 through STAT3 (Fig. 1D), and found that the extract of *Salvia officinalis* suppressed reporter expression induced by IL-6. Carnosol, luteolin and cirsiliol were identified as inhibitors of STAT3 signaling from *Salvia officinalis* (Fig. 1A, B). NMR spectra of these identified compounds were identical to those reported for carnosol, luteolin and cirsiliol [[13], [14], [15]]. Carnosol and luteolin showed clear dose-dependent suppression of luciferase activity (Fig. 1B). Cirsiliol dose-dependently suppressed luciferase activity in the dose range of 7.5–30 μM, but the suppression showed a plateau beyond 30 μM (Fig. 1C).

**Fig. 2:**
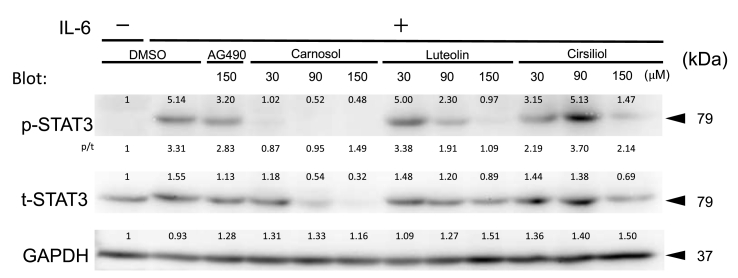
Inhibition of IL-6 dependent phosphorylation of Y705 of STAT3 by three compounds. Ten μg protein of each lysate was separated by SDS-PAGE. Y705-phosphorylated STAT3 (p-STAT3) and total STAT3 (t-STAT3) were detected by specific antibodies. GAPDH was used as a loading control for the Western blot. Relative intensity of bands compared to vehicle control was shown on each band after normalized by that of GAPDH band of each cell lysate. Ratios of p-STAT3 to total t-STAT3 (p/t) were given between the blots of p-STAT3 and t-STAT3. A result of one of the duplicate studies that gave similar results is shown.

Suppression of STAT3 phosphorylation at tyrosine Y705 induced by IL-6 contributes to the inhibition of STAT3 signaling [20]. As shown in Fig. 2, after serum starvation, phosphorylation of Y705 in STAT3 was induced by IL-6 stimulation. Carnosol, luteolin, and cirsiliol showed different patterns in suppressing STAT3 phosphorylation or STAT3 protein. Carnosol, at 30 μM, suppressed phosphorylated STAT3 induced by IL-6 strongly (about 80% suppression) but at a higher dose, carnosol suppressed not only phosphorylated STAT3 but also the total STAT3 level (Fig. 2). Cells treated with luteolin at 30 μM showed almost no change in the band intensity of phosphorylated and total STAT3 but at a higher dose luteolin clearly decreased phosphorylated STAT3 compared to total STAT3 (Fig. 2). HepG2 cells treated with cirsiliol at 90 μM did not show any clear decrease in the band intensities of phosphorylated and total STAT3 although there was apparent suppression of STAT3-responsive reporter expression (Fig. 1C). The results of STAT3 inhibitory activity in reporter expression by these three compounds were not in parallel with the changes in the levels of Y705 phosphorylation of STAT3, total STAT3 or their ratio (Fig. 1, Fig. 2).

**Fig. 3:**
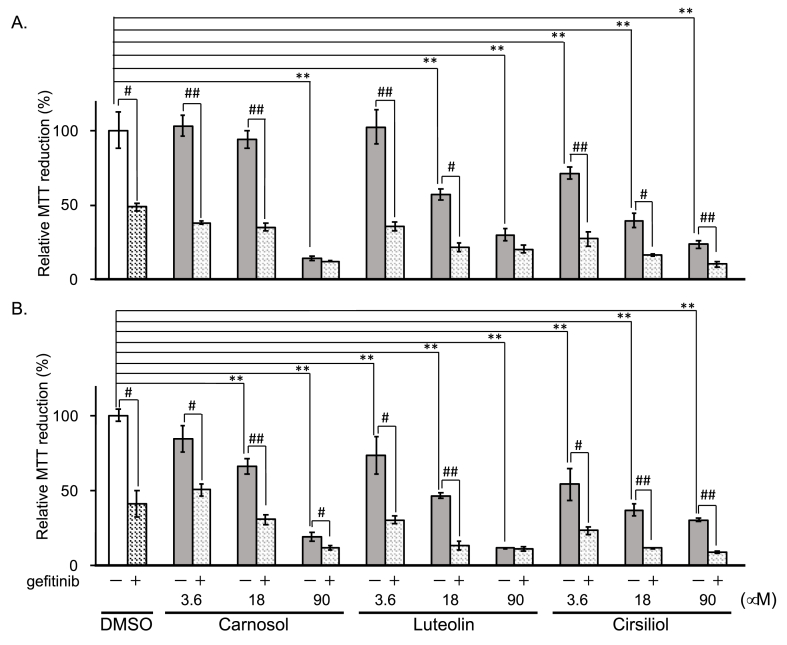
Additive anti-proliferative effect of three compounds with gefitinib on HepG2 (A) or A549 (B) cells using the MTT assay. Cells were treated with vehicle (open box) or each compound alone (gray box) or with 20 μM gefitinib (hatched box). The viability of control (vehicle treated cells) was regarded as 100%. Results are presented as means ± SD (n = 3). **p < 0.01 when compared with cells treated with vehicle alone by Dunnett's test. ^#^p < 0.05 and ^##^p < 0.01 when compared with cells treated with each compound alone by Dunnett's test.

Resistance to gefitinib (Iressa), a tyrosine kinase inhibitor of the epidermal growth factor receptor (EGFR) has been reported to occur in clinical trials [10]. Activation of STAT3 signaling in glioma and lung cancer has been suggested to be involved in the resistance to gefitinib [21,22]. In this study, the anti-proliferative activity of gefitinib against cancer cells was examined in the presence of carnosol, luteolin or cirsiliol by the MTT assay. Carnosol, luteolin and cirsiliol showed dose-dependent anti-proliferative activity against hepatoma HepG2 cells and lung cancer A549 cells and their anti-proliferative activity was enhanced by gefitinib, which is known to inhibit EGFR-dependent STAT3 activation but not IL-6-dependent STAT3 activation [23] (Fig. 3A and B). However, the anti-proliferative activity of these compounds described in Fig. 3 A and B was not in parallel with the level of phosphorylation of Y705 by these compounds at the dose of 90 μM in the serum-deprived condition described in Fig. 2. As compared to carnosol or luteolin, cirsiliol showed significant anti-proliferative activity in the MTT assay at 3.6 μM in both HepG2 and A549 cells (Fig. 3 A, B), but the suppression of phosphorylation of STAT3 by cirsiliol was weak even at 90 μM (Fig. 2), which suggests that cirsiliol has a unique anti-proliferative potential.

**Fig. 4:**
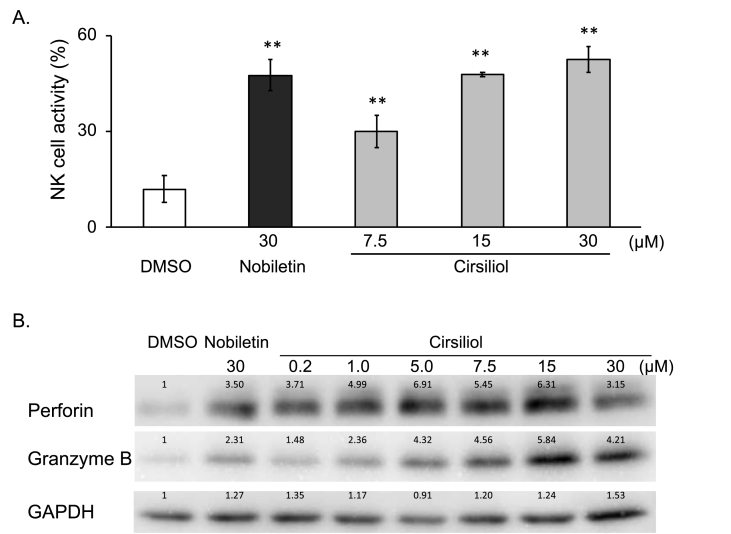
Activation of NK cells by cirsiliol. (A) Dose-dependent activation of cytolytic activity of KHYG-1 cells against K562 cells. Results are presented as means ± SD (n = 3). **p < 0.01 when compared with cells treated by vehicle by Dunnett's test. (B) Analysis of cytolytic compounds of KHYG-1 cells. Five μg of protein of each lysate was separated by SDS-PAGE. Perforin and granzyme B were detected by specific antibodies. GAPDH was used as a loading control for the Western blot. Relative intensity of bands compared to vehicle control is shown on each band after normalized by that of the GAPDH band of each cell lysate. A result of one of the duplicate studies that gave similar results is shown.

Polymethoxylated flavones including nobiletin have been reported to potentiate the cytolytic activity of naturel killer (NK) cells [12]. In this study, Cirsiliol, a dimethoxylated flavone enhanced the cytolytic activity of KHYG-1 NK cells concentration-dependently against erythroleukemia K562 cells (Fig. 4A) and induced perforin and granzyme B approximately concentration-dependently between 0.2 μM and 15 μM of cirsiliol for 24 h, suggesting that increase of NK activity was triggered by exocytosis of cytolytic granules by NK cells (Fig. 4B).

## 4 Discussion

In this study, several plant extracts were screened using the STAT3-responsive reporter system shown in Fig. 1D and found that the methanol extract of *Salvia* *officinalis* contained STAT3 signal inhibitory compounds and cirsiliol, luteolin, and carnosol were identified in the extract. Although the compounds reported as STAT3 signal inhibitory compounds [[3], [4], [5]], cirsiliol inhibited IL-6-dependent STAT3 reporter expression only in a partial manner since cirsiliol did not dose-dependently inhibit reporter expression beyond 30 μM (Fig. 1C and D), prompted us to compare the effect of these compounds on STAT3 protein with or without phosphorylation and the additive anti-proliferative effect to that of gefitinib which inhibits EGFR-dependent STAT3 activation.

Three STAT3 signal inhibitory compounds identified in *Salvia officinalis* exhibited an anti-proliferative effect on HepG2 cells and A549 cells and this anti-proliferative effect was enhanced by addition of gefitinib in a similar manner (Fig. 3A and B). However, the three compounds showed different patterns in dose-dependent suppression of STAT3 phosphorylation or total STAT3 protein quantity (Fig. 2), suggesting different mechanisms in inhibition of STAT3 signaling by the three compounds. Previous reports suggested that luteolin, carnosol and cirsiliol inhibited STAT3 signaling [[3], [4], [5],^24^]. Luteolin has been reported to promote the degradation of phosphorylated STAT3 in human hepatoma cells [24]. In our serum-starved condition, however, promotion of degradation of phosphorylated STAT3 by carnosol was more evident than luteolin (Fig. 2). Song et al. reported that luteolin stimulated STAT3 dephosphorylation by SHP-1 by protecting binding of phosphorylated STAT3 to heat shock protein 90 (HSP90) [5]. Besides HSP90, other heat shock proteins may also be involved in the regulation of STAT3. For example, Rocchi et al. showed that HSP27, a small HSP, directly interacted with STAT3 in prostate cancer cells and that suppression of HSP27 by anti-sense oligonucleotides suppressed the STAT3 level and expression of a STAT3 target gene, c-*fos* [25]. HSP27 may be a target of STAT3 signal inhibitory compounds. Carnosol has been reported to reduce DNA binding of STAT3 and attenuate phosphorylation of Janus-activated kinase-2 (Jak2) and Src kinase [4]. Very recently, Lim et al. reported that cirsiliol inhibited luciferase activity induced by phosphorylated STAT3 and speculated that cirsiliol regulated Jak2 phosphorylation [3]. In this study, phosphorylation of STAT3 at Y705 induced by IL-6 was weakly inhibited by AG490, a Jak2 kinase inhibitor, but not by cirsiliol at 90 μM in the serum-starved condition (Fig. 2). The inhibitory potential of STAT3 Y705 phosphorylation by each compound (Fig. 2) was not in parallel with the APRE-Luc reporter activity (Fig. 1 B, C) or anti-proliferative activity of each compound (Fig. 3). In particular, APRE-Luc reporter activity was not dose-dependently inhibited by cirsiliol beyond 30 μM (Fig. 1C), which suggested that cirsiliol inhibited only a partial pathway of STAT3 signaling. Since STAT3 inhibitors from natural sources have been reported to function through various mechanisms [7], the activation mechanism of STAT3 in each solid tumor needs to be considered before a STAT3 inhibitor can be developed for clinical use. Gefitinib (Iressa) is an inhibitor of EGFR-tyrosine kinase that inhibits EGFR-dependent but not IL-6-dependent phosphorylation of STAT3, and is clinically used to treat non-small cell lung cancer patients with EGFR-activating mutations [23]. However, gefitinib resistance has been observed. Inhibition of STAT3 signal by Static, a synthetic inhibitor of STAT3, increased the sensitivity of cancer cells to gefitinib [10]. We examined the additive effect of gefitinib on the anti-proliferative effect of carnosol, luteolin or cirsiliol in HepG2 and A549 cells. Gefitinib exhibited an additive anti-proliferative effect with the three compounds (Fig. 3A and B), although the mechanism of how the each compound inhibits STAT3 signaling seemed to be different (Fig. 2, Fig. 3). The nuclear PKM2, an isoform of muscle type of pyruvate kinase that is involved in a cancer-specific metabolism, activates nuclear STAT3 that correlates with gefitinib resistance in colorectal cancer cells [10]. Our preliminary study suggested that cirsiliol regulates PKM2 and that the additive anti-proliferative activity of cirsiliol to gefitinib may partly be explained by this PKM2 regulation (paper in preparation). However, whether nuclear PKM2 phosphorylates STAT3 or not is still controversial [26].

Lim et al. reported that cirsiliol suppressed the expression of IL-6-induced inflammatory marker genes, such as C-reactive protein (CRP), Interleukin-1β (IL-1β), intracellular adhesion molecule 1 (ICAM-1) and suppressor of cytokine signaling 3 (SOCS3) in Hep3B cells [3]. Down regulation of STAT3 targets, such as survivin, Cyclin-D1, -D2 and -D3 by carnosol in human colon cancer HCT116 cells and down regulation of survivin, myeloid cell leukemia sequence-1 (Mcl-1) and B-cell lymphoma-extra large (Bcl-xL) by luteolin in human gastric cancer cells, such as HGC27 cells has also been reported [4,5].

Cirsiliol, a dimethoxylated flavone, activated cytolytic activity of natural killer KHYG-1 cells against K562 cells (Fig. 4) like nobiletin, a polymethoxylated flavone [12], or luteolin, a flavone without methoxylation, whose NK activation was estimated by the MTT assay of K562 cells treated by none-adherent cells from isolated splenocytes [27]. NK cells utilize two mechanisms to kill the target cells; exocytosis of cytolytic granules or induction of apoptosis by death receptor-mediated pathway [28]. Induction of perforin and granzyme B supported partly the former mechanism of NK activation by cirsiliol. Since pre-treatment of Raji cells with naringenin, a kind of favanon, enhanced NK cell lysis activity by induction of NKG2D ligands, targets of NK cell activation receptor NKG2D [29], treatment of NK cells with cirsiliol and treatment of cancer cells with naringenin may simultaneously potentiate the anti-cancer activity of NK cells to cancer cells.

In conclusion, three compounds in *Salvia officinalis* showed direct anti-proliferative activity, while cirsiliol activated cytolytic activity of NK cells against cancer cells like luteolin [27] (Fig. 4). We identified three compounds as STAT3 signal inhibitory compounds, but the direct anti-proliferative activity was not in parallel with the inhibition of activation of STAT3 (Fig. 2, Fig. 3), suggesting that these three compounds in *Salvia officinalis* may also exert anti-proliferative activity on cancer cells through different mechanisms. Unknown multiple targets of each compound identified as an inhibitor of STAT3 signaling in this study may be involved in the putative anti-cancer property of *Salvia officinalis*.

## CRediT authorship contribution statement

**Maho Yanagimichi:** Investigation, Formal analysis. **Katsutoshi Nishino:** Investigation, Formal analysis. **Akiho Sakamoto:** Investigation, Formal analysis. **Ryusei Kurodai:** Investigation, Formal analysis. **Kenji Kojima:** Investigation. **Nozomu Eto:** Investigation, Methodology. **Hiroko Isoda:** Project administration. **Riadh Ksouri:** Resources, Writing - original draft. **Kazuhiro Irie:** Investigation, Writing - original draft. **Taiho Kambe:** Writing - original draft. **Seiji Masuda:** Writing - original draft. **Toru Akita:** Resources. **Kazuhiro Maejima:** Resources. **Masaya Nagao:** Supervision, Funding acquisition.

## Declaration of competing interest

The authors declare that they have no known competing financial interests or personal relationships that could have appeared to influence the work reported in this paper.

## References

[1] Poulios E., Giaginis C., Vasios G.K. (2020). Current state of the art on the antioxidant activity of sage (Salvia spp.) and its bioactive components. Planta Med..

[2] Jakovljević M., Jokić S., Molnar M., Jašić M., Babić J., Jukić H., Banjari I. (2019). Bioactive profile of various Salvia officinalis L. Preparations. Plants.

[3] Lim H.J., Jang H.J., Bak S.G., Lee S., Lee S.W., Lee K.M., Lee S.J., Rho M.C. (2019). In vitro inhibitory effects of cirsiliol on IL-6-induced STAT3 activation through anti-inflammatory activity. Bioorg. Med. Chem. Lett.

[4] Park K.W., Kundu J., Chae I.G., Kim D.H., Yu M.H., Kundu J.K., Chun K.S. (2014). Carnosol induces apoptosis through generation of ROS and inactivation of STAT3 signaling in human colon cancer HCT116 cells. Int. J. Oncol..

[5] Song S., Su Z., Xu H., Niu M., Chen X., Min H., Zhang B., Sun G., Xie S., Wang H., Gao Q. (2017). Luteolin selectively kills STAT3 highly activated gastric cancer cells through enhancing the binding of STAT3 to SHP-1. Cell Death Dis..

[6] Aggarwal B.B., Kunnumakkara A.B., Harikumar K.B., Gupta S.R., Tharakan S.T., Koca C., Dey S., Sung B. (2009). Signal transducer and activator of transcription-3, inflammation, and cancer: how intimate is the relationship?. Ann. N. Y. Acad. Sci..

[7] Wong A.L.A., Hirpara J.L., Pervaiz S., Eu J.Q., Sethi G., Goh B.C. (2017). Do STAT3 inhibitors have potential in the future for cancer therapy?. Expet Opin. Invest. Drugs.

[8] Yu H., Kortylewski M., Pardoll D. (2007). Crosstalk between cancer and immune cells: role of STAT3 in the tumour microenvironment. Nat. Rev. Immunol..

[9] Siveen K.S., Sikka S., Surana R., Dai X., Zhang J., Kumar A.P., Tan B.K., Sethi G., Bishayee A. (2014). Targeting the STAT3 signaling pathway in cancer: role of synthetic and natural inhibitors. Biochim. Biophys. Acta.

[10] Li Q., Zhang D., Chen X., He L., Li T., Xu X., Li M. (2015). Nuclear PKM2 contributes to gefitinib resistance via upregulation of STAT3 activation in colorectal cancer. Sci. Rep..

[11] Shou J., You L., Yao J., Xie J., Jing J., Jing Z., Jiang L., Sui X., Pan H., Han W. (2016). Cyclosporine A sensitizes human non-small cell lung cancer cells to gefitinib through inhibition of STAT3. Canc. Lett..

[12] Saito T., Abe D., Nogata Y. (2015). Polymethoxylated flavones potentiate the cytolytic activity of NK leukemia cell line KHYG-1 via enhanced expression of granzyme B. Biochem. Biophys. Res. Commun..

[13] Inatani R., Nakatani N., Fuwa H., Seto H. (1982). Structure of a new antioxidative phenolic diterpene isolated from rosemary (Rosmarinus-officinalis L). Agric. Biol. Chem..

[14] Yoshioka T., Inokuchi T., Fujioka S., Kimura Y. (2004). Phenolic compounds and flavonoids as plant growth regulators from fruit and leaf of Vitex rotundifolia. Z Naturforsch C J Biosci.

[15] Nagao T., Abe F., Kinjo J., Okabe H. (2002). Antiproliferative constituents in plants 10. Flavones from the leaves of Lantana montevidensis Briq. and consideration of structure-activity relationship. Biol. Pharm. Bull..

[16] Nakajima K., Yamanaka Y., Nakae K., Kojima H., Ichiba M., Kiuchi N., Kitaoka T., Fukada T., Hibi M., Hirano T. (1996). A central role for Stat3 in IL-6-induced regulation of growth and differentiation in M1 leukemia cells. EMBO J..

[17] Seiler-Tuyns A., Eldridge J.D., Paterson B.M. (1984). Expression and regulation of chicken actin genes introduced into mouse myogenic and nonmyogenic cells. Proc. Natl. Acad. Sci. U. S. A..

[18] Ohtera A., Miyamae Y., Nakai N., Kawachi A., Kawada K., Han J., Isoda H., Neffati M., Akita T., Maejima K., Masuda S., Kambe T., Mori N., Irie K., Nagao M. (2013). Identification of 6-octadecynoic acid from a methanol extract of Marrubium vulgare L. as a peroxisome proliferator-activated receptor γ agonist. Biochem. Biophys. Res. Commun..

[19] Nagahama K., Eto N., Shimojo T., Kondoh T., Nakahara K., Sakakibara Y., Fukui K., Suiko M. (2015). Effect of kumquat (Fortunella crassifolia) pericarp on natural killer cell activity in vitro and in vivo. Biosci. Biotechnol. Biochem..

[20] Inoue M., Minami M., Matsumoto M., Kishimoto T., Akira S. (1997). The amino acid residues immediately carboxyl-terminal to the tyrosine phosphorylation site contribute to interleukin 6-specific activation of signal transducer and activator of transcription 3. J. Biol. Chem..

[21] Lo H.W., Cao X., Zhu H., Ali-Osman F. (2008). Constitutively activated STAT3 frequently coexpresses with epidermal growth factor receptor in high-grade gliomas and targeting STAT3 sensitizes them to Iressa and alkylators. Clin. Canc. Res..

[22] Haura E.B., Sommers E., Song L., Chiappori A., Becker A. (2010). A pilot study of preoperative gefitinib for early-stage lung cancer to assess intratumor drug concentration and pathways mediating primary resistance. J. Thorac. Oncol..

[23] Bartolowits M.D., Brown W., Ali R., Pedley A.M., Chen Q., Harvey K.E., Wendt M.K., Davisson V.J. (2017). Selective inhibition of STAT3 phosphorylation using a nuclear-targeted kinase inhibitor. ACS Chem. Biol..

[24] Selvendiran K., Koga H., Ueno T., Yoshida T., Maeyama M., Torimura T., Yano H., Kojiro M., Sata M. (2006). Luteolin promotes degradation in signal transducer and activator of transcription 3 in human hepatoma cells: an implication for the antitumor potential of flavonoids. Canc. Res..

[25] Rocchi P., Beraldi E., Ettinger S., Fazli L., Vessella R.L., Nelson C., Gleave M. (2005). Increased Hsp27 after androgen ablation facilitates androgen-independent progression in prostate cancer via signal transducers and activators of transcription 3-mediated suppression of apoptosis. Canc. Res..

[26] Hosios A.M., Fiske B.P., Gui D.Y., Vander Heiden M.G. (2015). Lack of evidence for PKM2 protein kinase activity. Mol. Cell..

[27] Kilani-Jaziri S., Mustapha N., Mokdad-Bzeouich I., El Gueder D., Ghedira K., Ghedira-Chekir L. (2016). Flavones induce immunomodulatory and anti-inflammatory effects by activating cellular anti-oxidant activity: a structure-activity relationship study. Tumour Biol.

[28] Grudzien M., Rapak A. (2018). Effect of natural compounds on NK cell activation. J Immunol Res.

[29] Kim J.H., Lee J.K. (2015). Naringenin enhances NK cell lysis activity by increasing the expression of NKG2D ligands on Burkitt's lymphoma cells. Arch Pharm. Res. (Seoul).

